# Probing steps in DNA transcription using single-molecule methods

**DOI:** 10.1016/j.jbc.2021.101086

**Published:** 2021-08-14

**Authors:** Chun-Ying Lee, Sua Myong

**Affiliations:** 1 Department of Biophysics, Johns Hopkins University, Baltimore, Maryland, USA; 2 Physics Frontier Center (Center for Physics of Living Cells), University of Illinois, Urbana, Illinois, USA

**Keywords:** FRET, PIFE, transcription, magnetic tweezer, optical tweezer, atomic force microscopy, AFM, atomic force microscopy, CoSMoS, colocalization single-molecule spectroscopy, EC, elongation complex, FRET, fluorescence resonance energy transfer, ITC, initial transcription complex, Lk, linking number, PIFE, protein-induced fluorescence enhancement, RNAP, RNA polymerase, RPc, RNAP–promoter closed complex, RPo, RNAP–promoter open complex, smFRET, single-molecule FRET, smPIFE, single-molecule protein-induced fluorescence enhancement, TF, transcription factor, TIRF, total internal reflection fluorescence

## Abstract

Transcriptional regulation is one of the key steps in determining gene expression. Diverse single-molecule techniques have been applied to characterize the stepwise progression of transcription, yielding complementary results. These techniques include, but are not limited to, fluorescence-based microscopy with single or multiple colors, force measuring and manipulating microscopy using magnetic field or light, and atomic force microscopy. Here, we summarize and evaluate these current methodologies in studying and resolving individual steps in the transcription reaction, which encompasses RNA polymerase binding, initiation, elongation, mRNA production, and termination. We also describe the advantages and disadvantages of each method for studying transcription.

Transcription is the process by which the genetic code in DNA is transcribed to mRNA. This process involves a delicate balance of dynamic molecular interactions orchestrated by numerous *cis*- and *trans*-acting regulators. These regulatory elements influence each step of the transcription cycle, including promoter searching by RNA polymerase (RNAP), initiation, elongation, and termination (1, 2, 3, 4, 5). The transcription process has been extensively investigated and probed at multiple scales and using multiple methodologies, that is, from high-resolution structures of transcription complexes (6, 7, 8) to tracking stochastic transcriptional bursting in cells (9, 10, 11). Conventional biochemical, molecular, and structural analyses have deciphered the mechanism of transcription (6, 8, 12, 13) and revealed the extent of structural heterogeneity among molecules and complexes. However, determining the dynamic behaviors of individual molecules that contribute to the molecular heterogeneity requires advanced techniques with adequate temporal and spatial resolution.

Recent advances in single-molecule and single-cell techniques have provided a unique set of tools for visualizing the transcription process at increased molecular resolution (14). The first single-molecule experiment applied to transcription led to visualization of T7 RNAP transcribing RNA from a DNA template by capturing the Brownian motion of a gold particle tethered to the DNA strand (15). Later, an *in vitro* single-molecule study used fluorescence-based optical microscopy to probe fluorescently labeled RNAP and DNA during transcription to uncover kinetics and binding affinity (16, 17). Atomic force microscopy (AFM) (18), magnetic tweezers (19), and optical tweezers (20) resolved the mechanical force applied to DNA by RNAP and the resulting topological changes in DNA during transcription. Despite the diverse application of single-molecule methods in probing transcription, each tool has its own capacity of providing certain aspect of different stages. For example, fluorescence-based single-molecule detection with total internal reflection fluorescence (TIRF) microscopy can visualize binding and translocation movement of RNAP on the template DNA (21, 22, 23, 24, 25), and the concomitant structural changes within the DNA template (24, 26). Optical tweezers can measure the transcription-dependent mechanical force generated on DNA with a piconewton (pN) precision, and the corresponding changes in distance with the nanometer resolution, enabling the force–distance probing imparted by the activity of a single RNAP molecule (27, 28, 29). Magnetic tweezers are unique in their ability to apply the torsional constrain on the DNA to represent varying degree of supercoiled state of the DNA template. DNA melting can be measured by the torsional change that translates to the movement of the magnetic bead (30, 31). AFM allows for the measurements of the RNAP translocation on a DNA template and the concomitant changes in the architecture of the DNA. For instance, AFM image can deduce whether the DNA wraps around the RNAP (32, 33, 34, 35).

For cellular studies, different sets of techniques have been used to study transcription in live cells. One unique aspect of the live cell measurement is the visualization of single RNA transcript in real time to quantify the transcription process with a single molecule resolution. In fixed cells, single-molecule FISH can localize RNA to demarcate a particular sequence at the active site of transcription and the resulting transcript (36). Other methods, such as sequential FISH (seqFISH+) (37), multiplexed error-robust FISH (MerFISH) (38, 39), and *in situ* RNA-sequencing (40, 41, 42) enable whole-cell transcriptomic analysis. In addition, stem-loop labeling methods, such as MS2 and PP7 tags, are used to monitor the synthesis of mRNAs in live cells in real time (43, 44).

Although single-molecule imaging in live cells has become a popular tool in the field, *in vitro* single-molecule methods are still required to glean a detailed mechanism of molecular behavior. There are many previous papers offering comprehensive reviews on studying transcription in general (9, 45, 46, 47, 48, 49). Here, we summarize the methodologies of several key single-molecule assays for studying transcription mechanisms and review some advantages and disadvantages of each method.

## The kinetic cycle of transcription

Transcription can be divided into four main stages: promoter recognition, initiation, elongation, and termination. These steps are highly conserved across many species, but the details and level of complexity of each step varies across organisms. For example, the T7 bacteriophage RNAP is a single-subunit polymerase that initiates transcription without any transcription factor (TF); in contrast, prokaryotic and eukaryotic RNAPs are multi-subunit polymerases that require cofactors and TFs to initiate transcription (4, 50, 51). Owing to their simplicity, much of what we know about the various steps of transcription come from studies using the T7 system. However, recent studies using eukaryotic systems have deepened our understanding of gene regulation in higher-order organisms (20, 52, 53). In this section, we briefly summarize the current knowledge of each step in the transcription cycle and highlight the applications of single-molecule assays that have contributed to this knowledge.

### Promoter recognition

**Figure 1:**
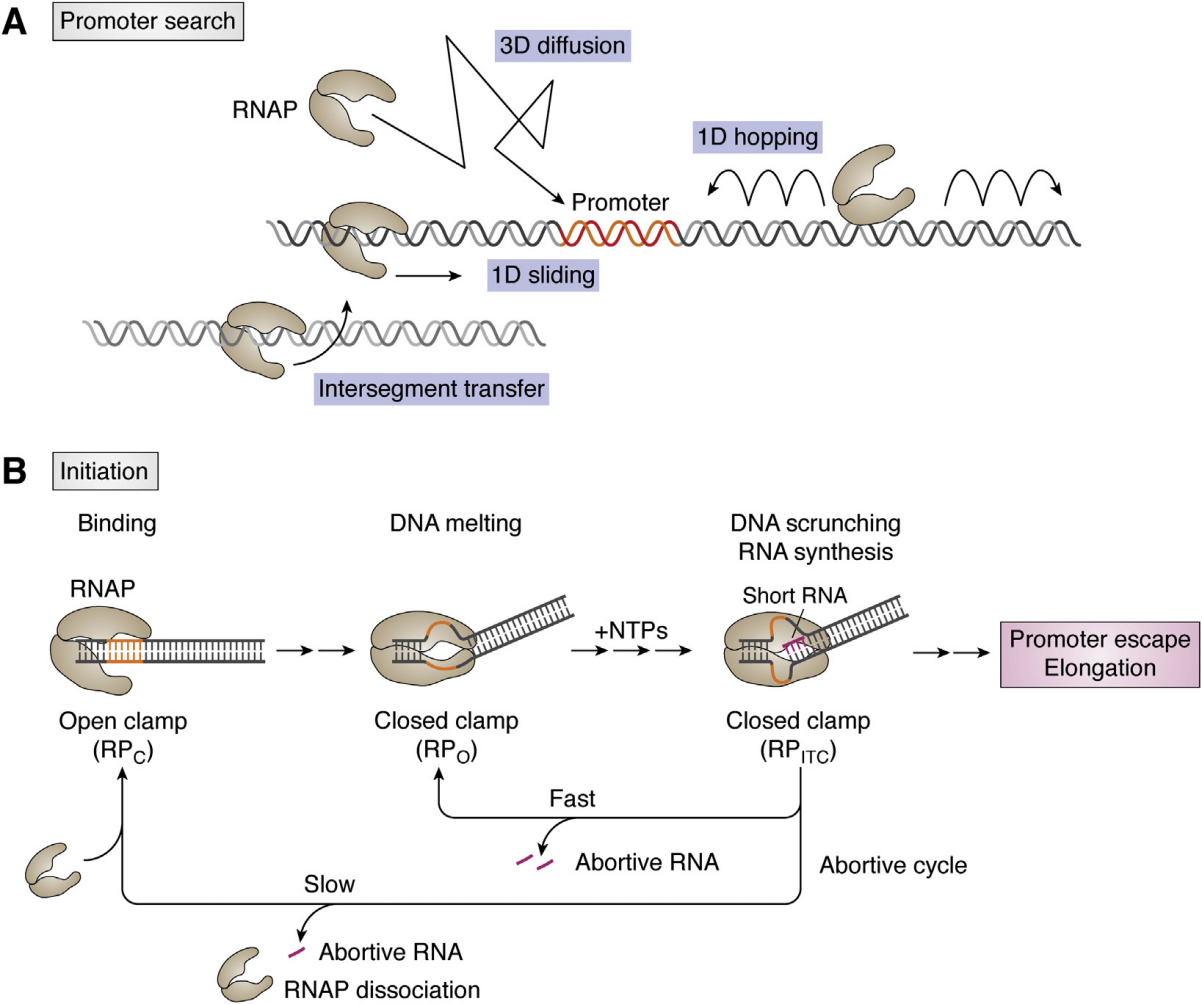
**The start of transcription.***A*, mechanisms of searching promoters by RNAP. *B*, the kinetic cycle of initiation. Upon RNAP binding to the promoter, the RNAP clamp closes to unwind DNA template. A short RNA is synthesized *via* a scrunching mechanism before RNAP escapes from the promoter region. The initiation complex (RP_ITC_) can enter either the elongation process or abortive cycle. RP_ITC_ returns to the RP_o_ cycle by releasing the short RNA, whereas full dissociation of RP_ITC_ requires a new RNAP to resume the cycle. RNAP, RNA polymerase; RPc, RNAP–promoter closed complex; RPo, RNAP–promoter open complex.

To initiate transcription, RNAP and TFs are recruited to the promoter (or other regions, such as enhancer), and RNAP undergoes a conformational change to unwind the DNA template and form an initiation complex. In other words, promoter recognition involves RNAP engaging with DNA and searching for the target sequence of the proper promoter. RNAP binding is concentration dependent, affected by both the local RNAP and DNA concentrations. For single-molecule study, a practical approach is to limit either the protein or the DNA to an extremely low concentration. For example, DNA can be immobilized on a glass surface *via* biotin-neutravidin conjugation for TIRF measurement (54) or tethered to a polystyrene bead trapped by a focused laser beam for optical tweezer experiment (55). In both cases, DNA concentration is fixed, whereas the RNAP concentration can be titrated to measure the binding kinetics. In addition, RNAP binding should be categorized as either non-specific or sequence-specific binding. Such tracking reveals an inherent binding kinetics of RNAP at or near the promoter (*k*_*app*_) that reveals how RNAP searches for a promoter. Recent single-molecule studies demonstrated that the search process mechanism includes one-dimensional sliding, three-dimensional direct binding (56, 57), hopping, and intersegment transfer (24, 33, 35, 58) (Fig. 1*A*). Also, the dynamics of promoter searching is influenced by the molecular crowding condition, chromatin structure, and concentration of the TFs (59, 60).

### Initiation

Upon RNAP (and other TFs) assembly at the promoter, several conformational changes take place before the formation of the initiation complex (Fig. 1*B*). First, RNAP binds the DNA template to form a pre-initiation complex. DNA is bent by the RNAP complex (clamp open) while remaining double-stranded (closed state), which is termed the RNAP–promoter closed complex (RPc). Next, the DNA template wraps around the RNAP as the RNAP clamp is closed, leading to unwinding and positioning of the DNA template to the RNAP active center. This new state is termed the RNAP–promoter open complex (RPo) and is ready to synthesize RNA (42, 61, 62, 63). The transition from the RPc to the RPo is a critical rate-limiting step in determining the initiation efficiency. It depends on the DNA sequence and the key regulators, such as the sigma factor that strongly stimulates the transition to the RPo state (62, 64, 65, 66). Furthermore, single-molecule fluorescence study demonstrated two intermediate RPc states including a short- and long-lived isomers, where only the long-lived RPc was capable of transitioning to the RPo state (42). After unwinding the DNA template at the RPo state, RNAP synthesizes and releases a short transcript (up to 11 nt) through a premature initiation cycle, called “abortive initiation” and the corresponding complex is termed the “initial transcription complex” (ITC or RP_ITC_) (67, 68) (Fig. 1*B*).

It was demonstrated by biochemical and single-molecule experiments that the abortive transcript is synthesized by a “scrunching mechanism,” which describes the RNAP remains stationary to the DNA template while the downstream DNA undergoes scrunching and compaction into the active center (69). If the RNAP aborts transcript synthesis and releases the compacted DNA template to downstream, the PR_ITC_ returns to the RPo state. Otherwise, RNAP escapes from the promoter by releasing the compacted DNA to upstream and entering the elongation step. Further single-molecule experiments revealed two abortive cycles, fast and slow, indicating returning to RPo state and fully dissociating from RPc, respectively (17, 70) (Fig. 1*B*).

### Elongation

**Figure 2:**
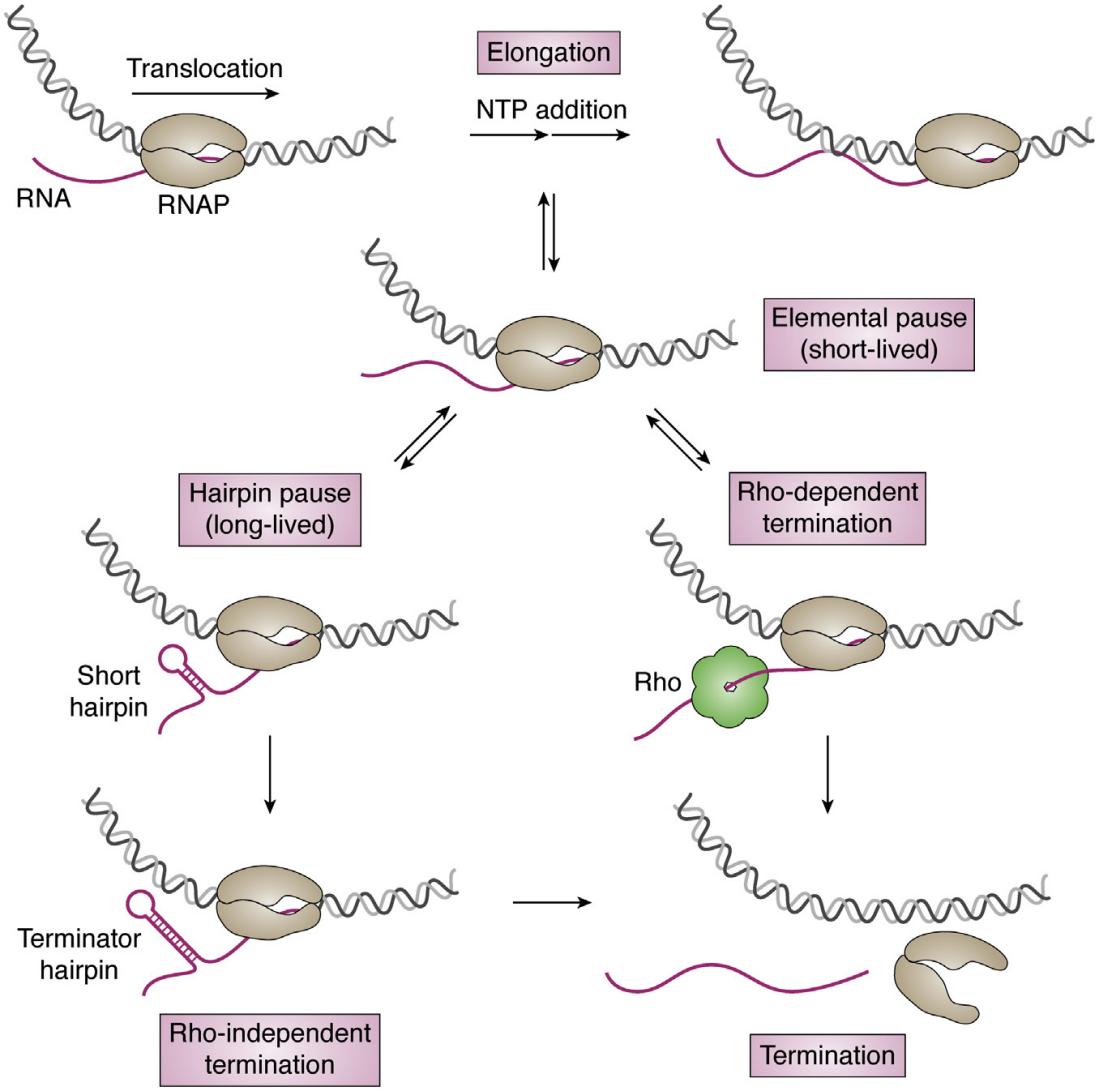
**The end of transcription.** Elongation process experiences short-lived pause events, which either revives to the next NTP addition or enters a long-lived pause state, for example, paused by a short RNA hairpin. Transcriptional termination (bacteria system, for instance) is conducted by either Rho-dependent mechanism or Rho-independent mechanism with the terminator hairpin.

Next, the RNAP escapes from the promoter and proceeds downstream, forming a “transcription bubble,” called the elongation complex (EC). The EC moves unidirectionally on the DNA template, synthesizing the nascent transcript and the reaction becomes a simple kinetic cycle of incorporating the appropriate ribonucleotide (NTP) into the growing chain of RNA from 5′ to 3′ direction (Fig. 2). Biochemical studies showed that the kinetic step was primarily regulated by the NTP concentration (25), indicating the rate of NTP incorporation as the main control of the RNAP movement and the resulting RNA synthesis. Nevertheless, a single-molecule study demonstrated that the kinetic cycle of elongation occurs through a Brownian ratchet rather than the power stroke mechanism (energy-dependent chemical step) (28). The Brownian ratchet model posits that the RNAP translocation is driven by the thermal noise but directed to move forward by NTP incorporation, which makes the RNAP movement sensitive to NTP concentration. Therefore, the incorporation of a single nucleotide can be described as a cycle that entails RNAP transitioning from the pre-translocated to post-translocated state, NTP binding, and formation of a phosphodiester bond on a nascent RNA chain (28). It was also shown that transferring to a post-translocated state can occur with or without the binding of incoming NTP (71).

In addition to on-pathway active translocation, the single-molecule study also demonstrated an off-pathway state called “pausing,” which has been observed in viral, prokaryotic, and eukaryotic RNAP (72, 73), albeit *via* different mechanisms (74). Generally, pausing is classified into short-lived (also termed “ubiquitous” and “elemental” pausing) and long-lived pausing (75, 76, 77) (Fig. 2). It is still not clear what causes short-lived pausing, but the long-lived pausing is initiated from the short-lived pausing with further backtracking step, caused by either random walks of the RNAP or trapping of other factors, such as an upstream RNA hairpin structure (78) or strong DNA:RNA hybrid (79). The RNAP pausing may be a mechanism to control the gene expression, especially in eukaryotic system, where several regulators and TFs are reported to pause or resume RNAP translocation (80).

### Termination

When the EC reaches a terminator sequence, the RNA transcript is released and the complex is dissembled (81, 82). In prokaryotic system, termination mechanism involves either the Rho-dependent or Rho-independent pathway (83, 84) (Fig. 2). The Rho factor, which is a ring-shaped helicase, moves along mRNA and pulls it out of the EC to trigger the conformational changes needed for termination (85). On the other hand, the Rho-independent termination is an intrinsic mechanism encoded in the RNA sequence. This pathway requires a post-transcriptional formation of an mRNA hairpin (usually GC rich) and a U-rich signal forming a weak hybrid within the RNAP active center (86, 87, 88, 89). Two models are used to explain the intrinsic termination: hyper-translocation of RNAP and shearing the DNA:RNA hybrid to force the RNA release and the dissociation of RNAP (90, 91). In addition, some TFs, such as NusA and NusG (92, 93), are also involved in the Rho-dependent termination but the mechanism remains unclear. In the eukaryotic system, the termination mechanism is more complicated because of the additional steps of post-transcriptional processing, such as RNA polyadenylation (84). Moreover, termination also involves RNA polymerase II undergoing dephosphorylation of C-terminal domain and detachment from the TFs (94). To date, researchers have applied optical tweezers to measure the terminator hairpin (95) and riboswitch structure (96) as well as the binding and translocation of the Rho factor (97). In addition, magnetic tweezers are also applied in monitoring the disappearance of transcription bubble in helicase-induced termination (98).

## Single-molecule methods in transcription studies

In this section, we discuss the application of single-molecule tools to probe the transcription cycle. The methods discussed fall into three major categories: fluorescence-based method, force-based assay, and AFM.

### Fluorescence-based assays

The fluorescence-based assays are aimed at directly visualizing individual molecules. Typically, DNA is fluorescently labeled *via* chemical conjugation or by intercalating dye, and RNAP (or other cofactors) is labeled chemically or bound by a fluorescently labeled antibody. Single-molecule fluorescence detection performed by TIRF microscopy is an ideal method that provides high spatial (nanometer, nm) and temporal (minisecond, ms) resolution with a relatively high signal-to-noise ratio (54). The use of multicolor fluorescence expands the applicability of these experiments. Overall, the fluorescence-based assays described here offer complementary methods to visualize molecular interactions at the single-molecule level.

#### DNA curtain

**Figure 3:**
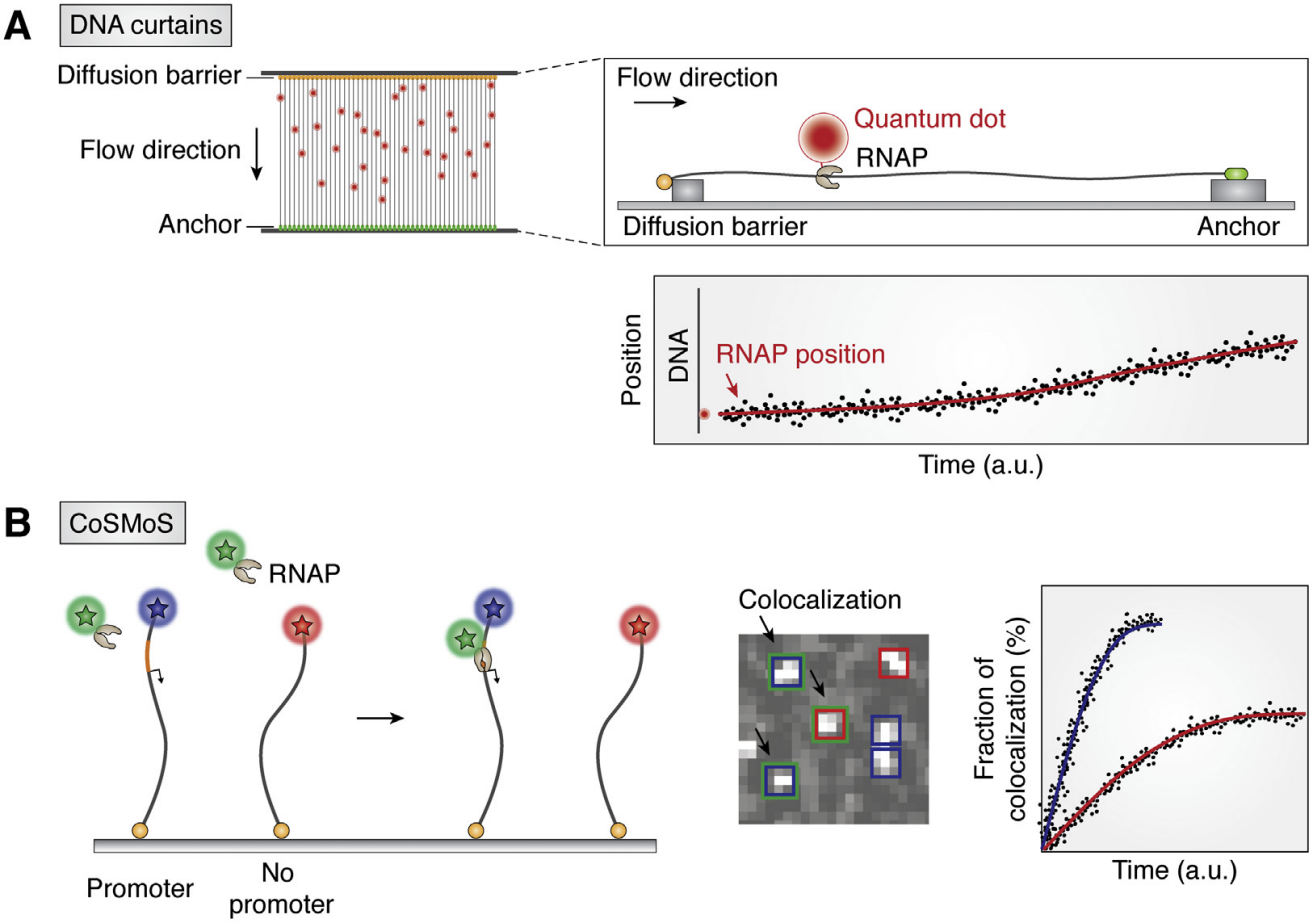
**Fluorescent-based assays.***A*, DNA curtain. DNA curtain creates a 2D array by aligning DNA molecules with the diffusion barrier and anchor. Translocation of the quantum-dot (Qdot)-tethered RNAP is tracked by single-molecule fluorescence microscopy. *B*, CoSMoS. CoSMoS distinguishes the binding *via* colocalized signals. For example, *blue* and *red squares* indicate the position of the DNA molecule with and without a promoter, and the *green squares* indicate the binding of RNAP. Therefore, the two-color squares (*green*/*blue* or *green*/*red*) indicate the binding of RNAP on the DNA strand. The fraction of bound DNA can be plotted as a function of time to evaluate the kinetics. The schematics of DNA curtains and CoSMoS are inspired by the work of Wang (58) and Friedman (24), respectively. CoSMoS, colocalization single-molecule spectroscopy; RNAP, RNA polymerase.

The DNA curtain assay is performed by aligning DNA molecules on a surface (58, 99) (Fig. 3*A*, left). The DNA molecules are tethered on one end and stretched by a hydrodynamic flow to create a linear array to which RNAP can be applied (Fig. 3*A*, right top). The array of DNA molecules is visualized by an intercalating YOYO dye to confirm the proper immobilization and linear configuration. The RNAP can be tagged with a quantum-dot, a bright fluorescent nanoparticle, for tracking the movement. The main advantages of the DNA curtain platform are that long DNA segments, for example, λ DNA (about 48 kb), can be imaged and long-range movement of DNA-binding proteins can be probed (58). The extended length of DNA allows for capturing high-speed sliding or three-dimensional diffusion events. The DNA curtain platform has been used to investigate RNAP binding and promoter search on long DNA templates. The movement of RNAP was analyzed in the form of a kymograph, a plot of the position over time (Fig. 3*A*, right bottom). In the absence of NTP, RNAP stays at a promoter for a short time (∼sec) until it randomly steps out or dissociates from the promoter. On the other hand, in the presence of NTP, RNAP synthesizes RNA and moves away from the promoter, which can be measured up to a long-distance range (∼μm) (58). One downside of this experiment is that the native context for DNA flexibility is absent in a stretched DNA configuration, that is, the potential search mechanism, such as segmental transfer with DNA looping, may be limited and suppressed (32).

#### Colocalization single-molecule spectroscopy

Colocalization single-molecule spectroscopy (CoSMoS) (24, 100) uses multi-color fluorescence to track complex molecular events. CoSMoS was used to investigate the kinetics of RNAP binding on DNA with or without a promoter. One end of DNA was immobilized on the surface, allowing the rest of the DNA to be freely flexible in solution (Fig. 3*B*, left). DNA and RNAP were labeled with different fluorophores such that binding of RNAP was read out as a colocalization of both signals within the same diffraction-limited spot defined by the position of the DNA (Fig. 3*B*, middle). The duration of the colocalized signal is interpreted as the lifetime of binding (except when it is photobleached). This assay can be extended to a three-color laser system, for example, promoter-containing DNA and the control DNA without a promoter can be labeled with different fluorophores, and the protein with the third color. Therefore, the real-time detection of colocalization patterns reflects the relative binding affinities of RNAP to either substrate in the same condition (Fig. 3*B*, right). However, CoSMoS is not ideal for detecting one-dimensional sliding because all the signals are collected from a confined imaging area, which cannot provide long-range position changes. CoSMoS has been applied to study the preinitiation complex assembly process of the yeast system performed with combinations of labeled DNA, RNAP, and TF (TFIIE and TFIIF) (101). It is worth noting that both the DNA curtain and the CoSMoS are limited in terms of the concentration of fluorescently tagged molecules because of the increased background fluorescence. TIRF imaging is built for a selective excitation in an evanescent field (∼200 nm from surface), and the intensity in the evanescent field decays exponentially as a function of distance away from the slide surface. Therefore, a relatively high concentration (higher than 5–10 nM) of diffusive molecules can be excited in the evanescent field and make the signals indistinguishable.

#### Single-molecule fluorescence resonace energy transfer

The single-molecule fluorescence resonance energy transfer (smFRET) is a widely applied single-molecule technique that depends on the measurement of the distance between a pair of fluorophores (donor and acceptor) attached to the molecules of interest. Fluorescence resonance energy transfer (FRET) occurs *via* a non-radiative energy transfer from a donor (high-energy state, shorter excitation wavelength) to an acceptor (low-energy state, longer excitation wavelength). FRET efficiency depends on the distance between the donor and acceptor with a sixth-order reciprocal relationship, thus providing an extremely sharp distance dependence. FRET displays a distance sensitivity between 3 and 8 nm; therefore, it is suited to detect short distance changes between the two interacting molecules or two sites within one molecule (102). For example, smFRET has been applied to visualize the opening of the transcription bubble and the dynamics within an RNAP clamp.

Upon recognition of a promoter, RNAP undergoes a series of conformational changes to form an initiation complex, which entails a structural transition from a closed state (RPc) to an open state (RPo), while also unwinding the DNA to form a transcription bubble (Fig. 1*B*) (103, 104, 105). Compared with a previous study that used 2-aminopurine enhancement to measure the initiation rate and estimate the size of transcription bubble (64, 106), smFRET can directly detect the structure–function relationship underlying the dynamics of the transcription initiation in real time.

**Figure 4:**
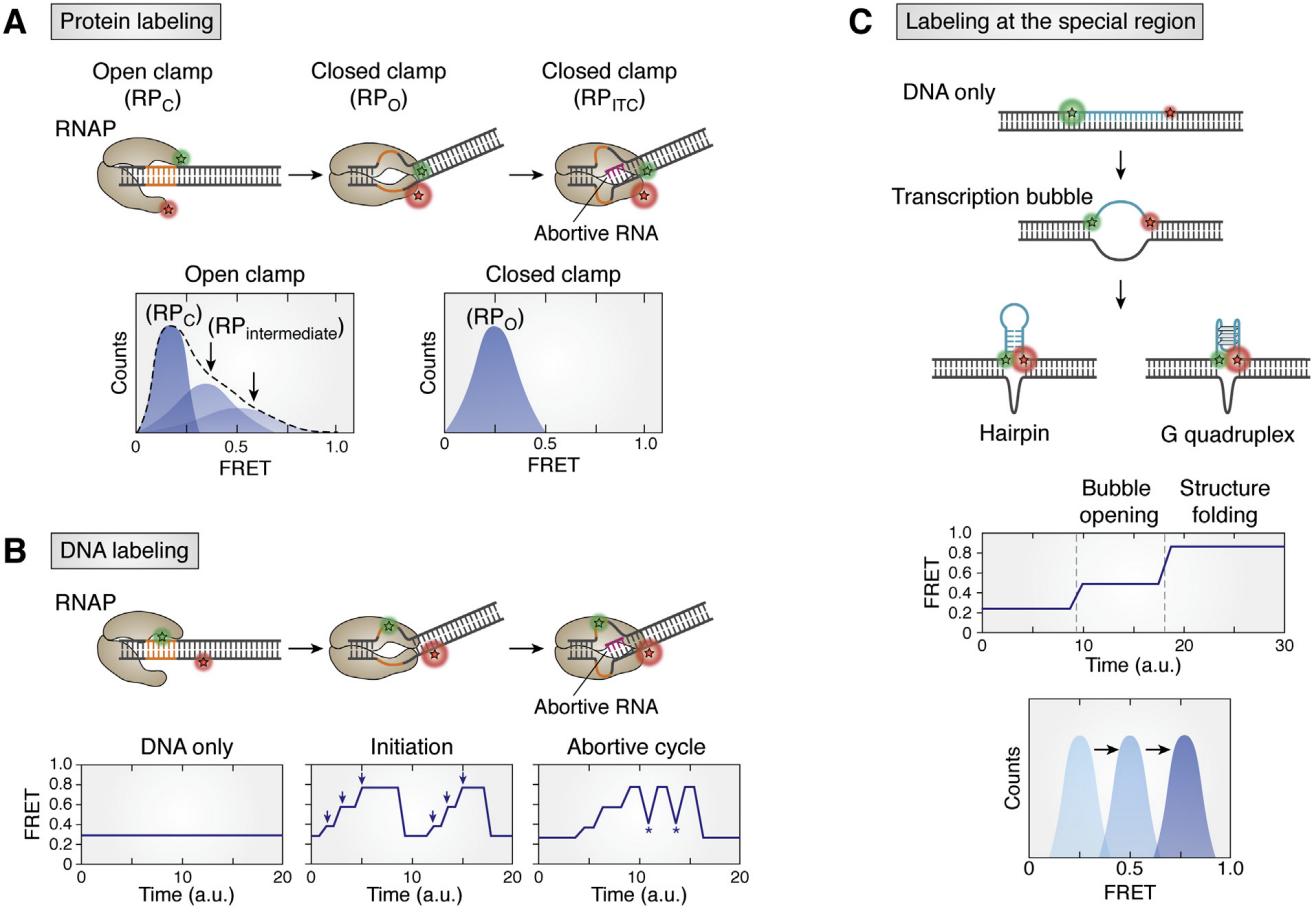
**Applications of the smFRET.***A*, smFRET with protein labeling. Clamp closing as unwinding the DNA increases the FRET efficiency. In addition, two more FRET populations (*black arrows*) appear after RNAP binding, indicating intermediate states (RP_intermedicate_) in transition between RP_c_ and RP_o_. The scheme is inspired by Chakraborty’s work (62). *B*, smFRET with DNA labeling. Bending and unwinding the DNA cause stepwise transitions of the FRET state (*blue arrow*). After RNAP leaves the promoter-proximal region, FRET transits to the original value, indicating the duplex structure. Abortive cycle is observed in several transitions (*asterisk*) between two FRET states but not the duplex states. The scheme is inspired by Koh’s work (30). *C*, smFRET can be used to detect interesting sequence, which folds into other structures. Examples are depicted from our previous work of other secondary structures, for example, hairpin and G-quadruplex (111, 117). The FRET efficiency increases after formation of transcription bubble and folding of structure. Stepwise formation can be observed through FRET trajectory. The FRET histogram can demonstrate the overall distribution of each structure state. The scheme is recreated from our previous work (111). RPc, RNAP–promoter closed complex; RPo, RNAP–promoter open complex; smFRET, single-molecule FRET.

Previous studies using smFRET have revealed various aspects of dynamics during the transcription initiation and transition states (17, 26, 31, 62, 69, 70, 107, 108, 109, 110, 111). Typically, to visualize the conformational changes during initiation, FRET-pair dyes, such as Cy3 and Cy5, can be attached to RNAP subunits (Fig. 4*A*) or on DNA template surrounding the promoter (Fig. 4*B*). For the first labeling scheme, RNAP subunits are labeled with FRET dyes, that is, the donor on β lobe tip and the acceptor on β′ clamp tip (62). Thus, opening and closing of the RNAP clamp was monitored by FRET decrease and increase, respectively (Fig. 4*A*). By stalling RNAP at a particular DNA sequence, each initiation state was captured at different positions along the transcript. These FRET states were further examined and validated using transcription inhibitors. In addition, the FRET values were used to estimate the change of the clamp size (10–20 Å) in agreement with the crystal structures.

When the dye pair is positioned upstream and downstream of the T7 RNAP promoter (Fig. 4*B*), it is suited for probing DNA melting and bubble formation involved in the initiation process (17, 26, 111). This strategy has been cleverly applied to examine the stepwise progression of the early-stage transcription by halting the reaction with limiting the NTP content. Furthermore, it has been shown that RNAP synthesizes early transcripts *via* a “scrunching mechanism” (69). A recent smFRET study measured continuous RNAP progression with all NTPs present, demonstrating the sequence of events, including the transcription bubble formation and progression through the DNA template (17, 26). The transient increase of FRET efficiency corresponds to the bubble formation, and the subsequent FRET decrease indicates reannealing of the DNA after the bubble passes through the FRET-labeled segment (Fig. 4*B*, middle). This experimental scheme provides a unique opportunity to detect abortive initiation, which produces short and incomplete transcripts before RNAP escapes from the promoter and enters the elongation phase (112, 113). Unlike the single FRET event that signifies successful and normal rounds of initiation, the abortive initiation gives rise to characteristic FRET fluctuations (Fig. 4*B*, right).

Moreover, the transition from the initiation to the EC can also be distinguished by adding labeled RNAP with a labeled DNA template, but it requires complicated analysis of the FRET signals (17, 114). Similarly, labeling the sigma factor and TFs can be applied to track the intermolecular interactions during the initiation complex formation in the bacterial and the eukaryotic system (22, 115). We note that while the smFRET is optimal for measuring the kinetic parameters and the mechanistic details involved in the transcription initiation, it is blind to mechanical force and accurate topological changes involved in the DNA melting.

Another application of smFRET is to capture transient formation of DNA secondary structures during transcription. Such structures include the hairpin, pseudoknot, G-quadruplex, i-motif, and R-loop (111, 116, 117, 118). The FRET-paired dyes can be located across the particular sequence element but within a FRET-sensitive distance, so the formation of expected structure will change the FRET signal as a function of the transcription reaction (Fig. 4*C*). For example, the formation of a transcription bubble unwinds a part of ssDNA on the non-template strand. The released region can form a stable secondary structure such as G-quadruplex or hairpin (111, 117), and the two dyes located at either end of the sequence on the non-template strand will show a prominent increase in FRET during the transcription reaction (Fig. 4*C*). This assay can correlate the cotranscriptional structure formation during the transcription process.

#### Single-molecule protein induced fluorescence enhancement

Protein induced fluorescence enhancement (PIFE) is a single-color method that offers a unique and simple approach to study molecular interactions. The fluorescence enhancement effect induced by a proximal protein binding occurs in cyanine dyes, such as Cy3 and Cy5, all of which consist of symmetric carbocyanine structures. The cyanine dyes store and release photon energy at a particular wavelength while undergoing *cis*-*trans* isomerization, which entails interconversion between the two configurations *via* the central carbon-carbon double bonds. Binding of a viscous molecule such as protein stabilizes the photoactive *trans* state, thereby increasing the quantum yield and lifetime of the dye, making the fluorescence signal 2 to 3 folds brighter than by itself (119, 120). Furthermore, the increase in the PIFE signal is linearly correlated to the distance between the protein and the dye within 0 to 3 nm (119, 121, 122). Because PIFE is sensitive to a short-distance change where FRET is insensitive, PIFE can complement FRET for obtaining the entire distance range between 0 and 8 nm. In general, PIFE is a convenient tool for capturing protein–nucleic acid interaction at the single molecule level (17, 111, 123, 124, 125).

**Figure 5:**
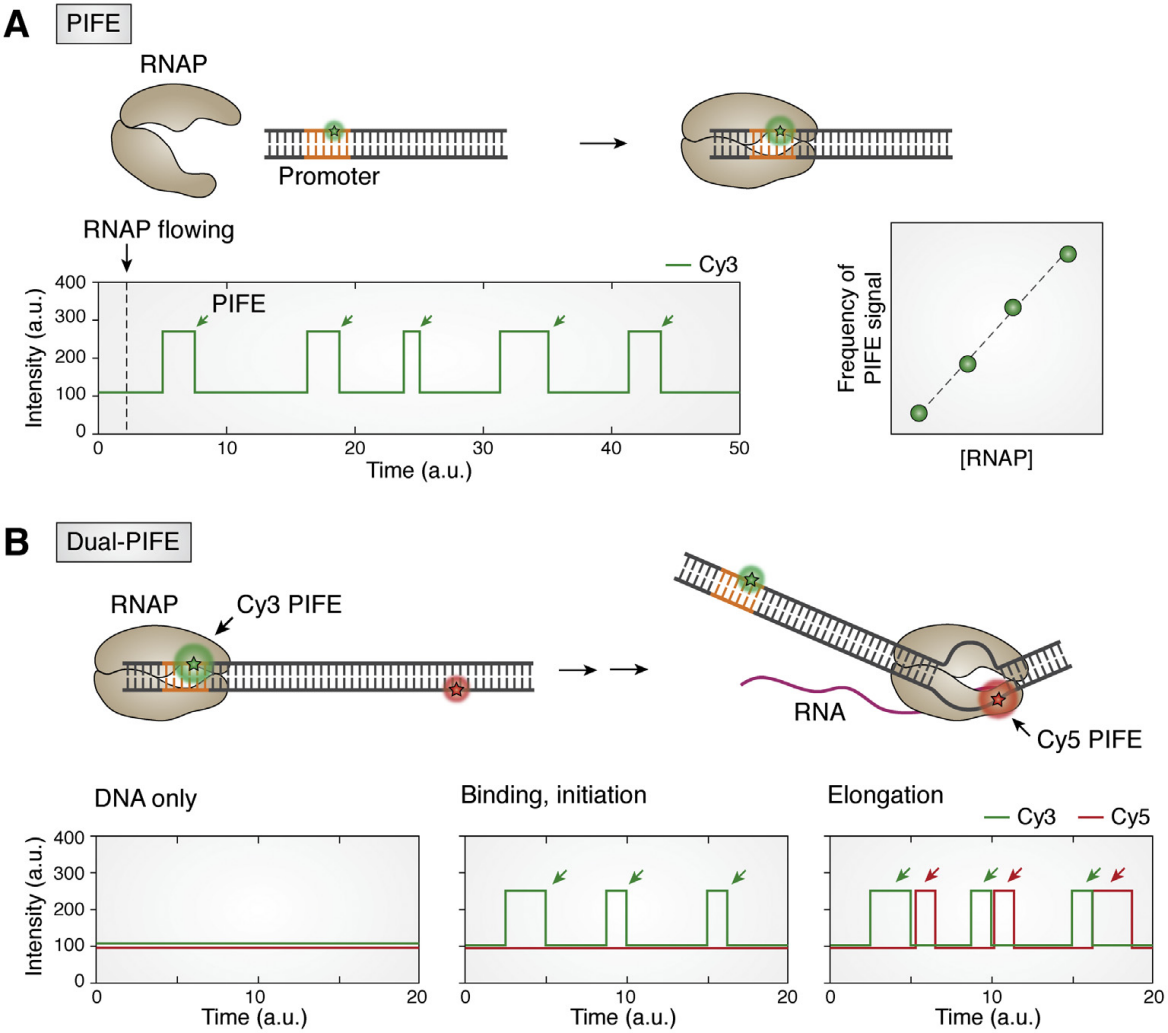
**Applications of smPIFE.***A*, smPIFE detects RNAP binding. PIFE signal is 2- to 3-fold higher than initial intensity. The *green arrow* indicates the PIFE events. The frequency of PIFE signals is RNAP concentration dependent. *B*, successful elongation event is observed by dual PIFE system. Protein binding and initiation only cause single PIFE signal (*green arrow*). Dual PIFE signal appears when RNAP moves downstream and induces the PIFE signal of the second dye (*red arrow*). PIFE, protein induced fluorescence enhancement; RNAP, RNA polymerase; smPIFE, single-molecule protein induced fluorescence enhancement.

Single-molecule protein-induced fluorescence enhancement (smPIFE) was applied to study the RNAP binding and movement in our recent study. A single fluorophore located at the promoter site showed a sudden spike of fluorescence intensity, reporting on the specific binding of RNAP *via* PIFE (Fig. 5*A*, top). The PIFE signal was observed immediately after the addition of RNAP, indicating that the signal represents RNAP binding (Fig. 5*A*, bottom left). Importantly, in the PIFE experiment, DNA is labeled, but not the protein. Therefore, the validity of the PIFE signal can be tested by titrating RNAP concentration because the RNAP binding frequency should scale with the RNAP concentration (Fig. 5*A*, bottom right). Furthermore, since PIFE is extremely sensitive to the local environment, the level of enhancement can distinguish between the RPc and RPo state of binding complex in the presence of NTP (17, 23). However, smPIFE is unable to resolve position information such as the movement expected from the one-dimensional sliding because the signal disappears once RNAP leaves away from the dye.

The smPIFE experiment can also be used to study elongation, albeit within a limited length. We recently presented a novel dual-color PIFE (dual PIFE) assay to efficiently monitor multiple rounds of successive elongation cycles (111). For dual PIFE experiment, unlike the smFRET design, the two dyes (Cy3 and Cy5, for example) are positioned on the DNA template but far apart from each other, that is, outside of the FRET-sensitive distance range, so that each dye can independently undergo PIFE without any interference of the FRET signal (Fig. 5*B*). Because the PIFE signal is observed when RNAP moves across the dye, the sequential PIFE signals from each dye in succession from the same single-molecule spot reports on the movement of RNAP on the DNA from the first to the second dye. Unlike the alternating-laser excitation (126), dual PIFE measurement requires continuous excitation of both dyes to capture the PIFE events from both channels simultaneously.

In our experimental design, the first dye (Cy3, green) is labeled at the promoter, near the RNAP-binding site, and the second dye (Cy5, red) is labeled 40 base pairs away from the first dye. Therefore, the first PIFE signal turns up when RNAP passes through the promoter (Fig. 5*B*, bottom middle), and the second PIFE occurs if the same RNAP arrives at the 40th base pair downstream, resulting in the green and red signals appearing in tandem succession in the single-molecule time trajectory (Fig. 5*B*, bottom right). That is, the tandem dual PIFE signal appears only if the elongation was successful up to the second position. In the case of the failed elongation, we only obtain the first green PIFE without the second red PIFE event. We note that the failed elongation can be distinguished from photobleaching of the second dye because we can monitor both dye signals continuously because of the continuous illumination by both lasers. In this manner, the fraction of successful elongation events can be calculated over the total attempted elongation. Furthermore, the dwell time of each PIFE signal and the time interval between two PIFE signals can be used to quantify the overall speed of RNAP across the two dye positions. Taken together, the dual PIFE signals works as a “molecular gate” to monitor the entry and the exit of the RNAP along particular positions on the DNA template. For example, a previous study temporarily decoupled PIFE and FRET to study RNA release and folding (127). We envision that a multi-color PIFE assay should be feasible, and such capability will enable the detection of more complex observables in one platform.

#### Fluorescent probe

Another fluorescent-based assay was developed for probing the synthesis of mRNA, which is the final step of the transcription process, that is, how many transcripts are produced by RNAP per DNA molecule per unit time. Single-molecule methods have led to detection of stochastic mRNA transcription both *in vitro* and *in vivo* (11, 14, 43, 128, 129, 130). For real-time single-molecule detection of *in vitro* transcription, the basic concept is to visualize mRNA cotranscriptionally, that is, while the RNA is being transcribed. One way is to incorporate fluorescently modified NTP to light up the RNA molecule (131). Another method, similar to FISH, is to hybridize fluorescently labeled oligonucleotides to a transcript (128, 129). Although both strategies lead to the direct visualization of RNA products, both suffer from extremely high fluorescence background, preventing observation of multiple rounds of transcription. As mentioned above, the detection under a TIRF microscope typically accommodates up to 10 nM of fluorescent molecules, which is far below the requirement for efficient DNA/RNA hybridization for detecting mRNA. Therefore, both methods are not optimal for monitoring the real-time RNA transcript.

**Figure 6:**
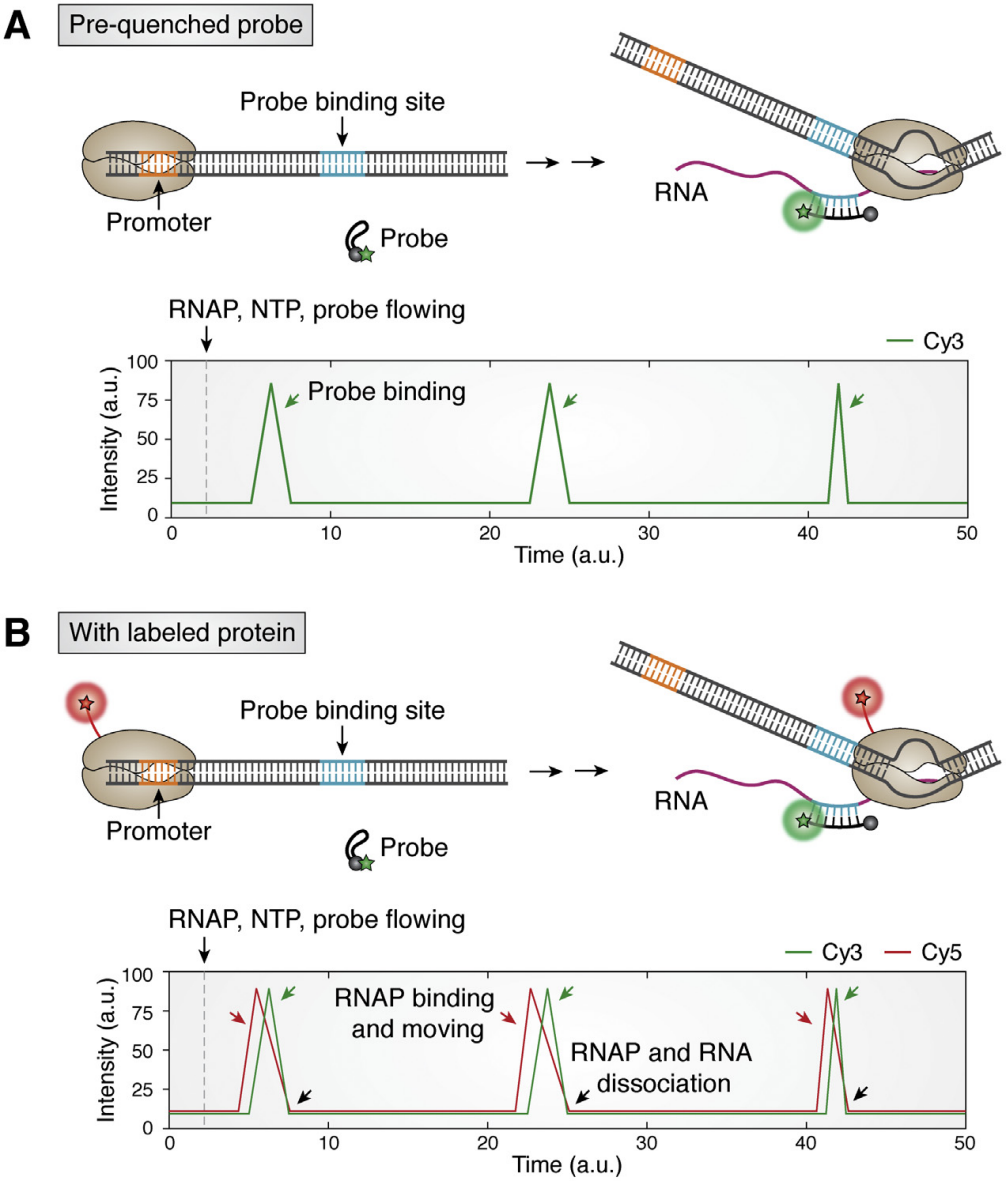
**Transient RNA production is visualized by a prequenched probe.***A*, RNA transcript is detected by a complementary probe to RNA sequence (colored in *magenta*). The fluorescence signal appears when probe anneals with RNA transcript (*green arrow*). *B*, coupling with labeled protein, binding of RNAP and RNA production are visualized together to determine the timing of beginning and dissociation of transcription. RNAP entering, probe binding, and dissociation are indicated by *red*, *green*, and *black arrows*, respectively. The scheme is inspired by Zhang’s work (132). RNAP, RNA polymerase.

To overcome this limitation, we and others developed a molecular beacon, which is a prequenched probe that fluoresces only upon hybridizing with the complementary mRNA product (132) (Fig. 6*A*). The key difference from previous methods is that the prequenched probe is initially dark and becomes dequenched and fluorescent only upon hybridizing to the RNA molecule. This strategy reduces the intrinsic background drastically, allowing up to 500 nM probe concentration to be added to single-molecule transcription assays. This concentration is sufficient to induce instantaneous annealing to the nascent RNA strand in a cotranscriptional manner. More importantly, the sequence and length of a probe should be carefully designed based on the calculation of the folding and annealing energy. That is, the hybridization rate of the probe to a target mRNA exceeds the diffusion rate, and the probe position should not be far from the promoter to ensure that the probe binds before the mRNA detaches from the DNA template. Moreover, single molecular dynamics of protein–DNA interactions, for example, RNAP, and TF can be simultaneously detected by combining labeled protein and prequenched beacon. The signal from the protein indicates the initiation of transcription, while the beacon signal reflects the RNA synthesis (Fig. 6*B*). One round of dual signal likely indicates a single TF-induced single turnover reaction. However, as stated above, only a low concentration of labeled protein can be applied to avoid high fluorescence background. Also, the advantage of using the quencher probe can be extended to a long transcript, that is, above 100 nucleotides as long as the probe-binding site is available, and the probe sequence is optimized.

### Force-based assays

#### Optical tweezers

Recent single-molecule studies on elongation have mostly focused on the transition from initiation complex to the early EC (23, 133). As stated above, while the structural dynamics of protein and DNA can be observed by directly labeling RNAP or DNA (17, 21, 121, 122), FRET detection is limited to approximately 3 to 8 nm distance, far below the distance range for elongation. One single-molecule method to measure elongation process is the optical tweezers, which is suited to measure a large distance change within a tethered molecule or between two tethered molecules (20, 134).

**Figure 7:**
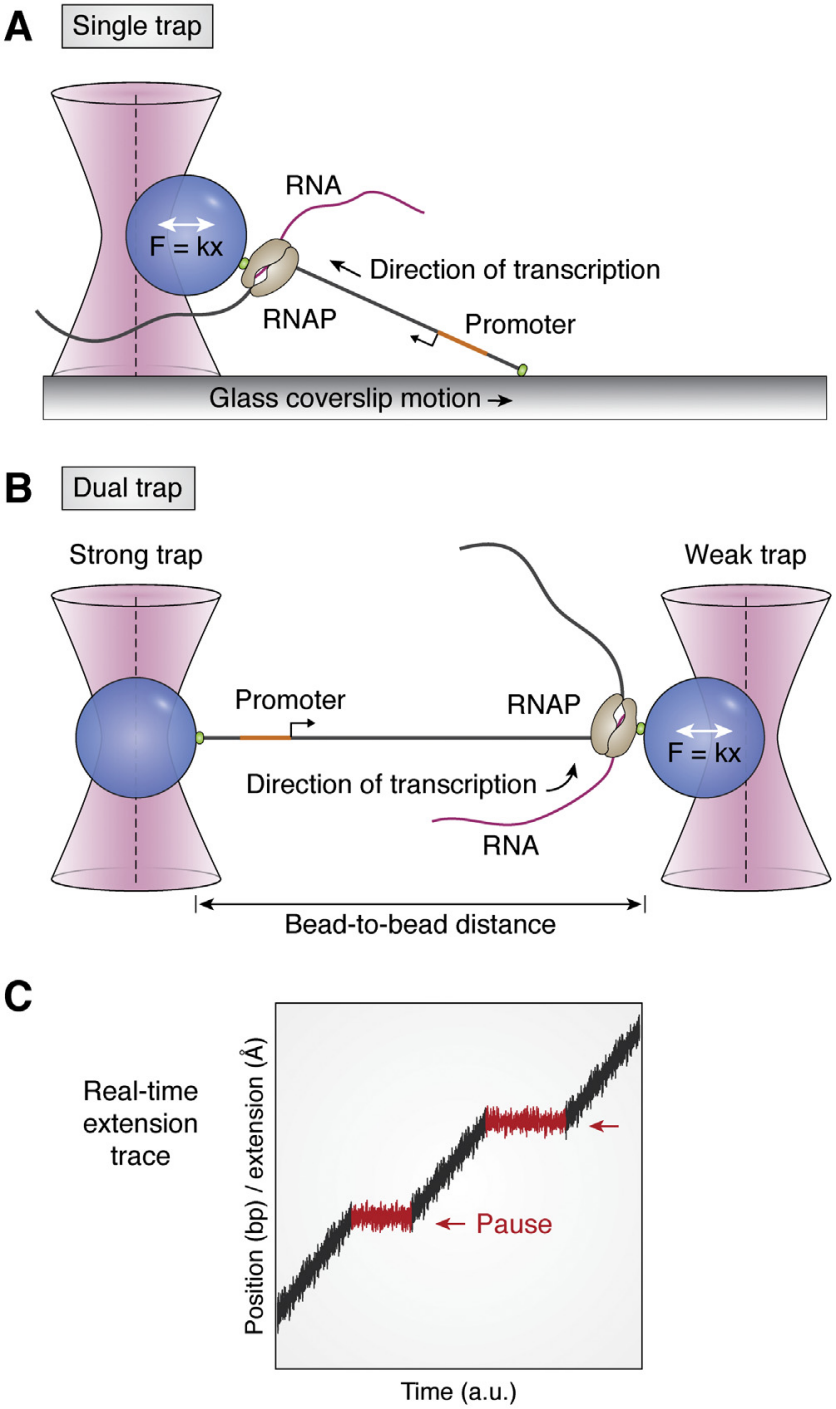
**RNAP translocation detected by optical tweezers.***A*, single optical trap system. One end of the DNA strand is immobilized on the slide surface, and the other end is dragged by the RNAP, which is tethered to the bead. *B*, dual optical trap system. One end of the DNA strand and RNAP are tethered to the beads. When RNAP moves to the other end of the DNA strand during elongation process, it causes the tension force change (ΔF), which can be further interpreted to the coverslip displacement (single trap) or the change of bead-to-bead distance (dual trap). *C*, an example of the real-time extension trace. DNA extension is plotted as a function of time. The pause events (*red arrow*) are observed as temporary stops with certain time intervals. RNAP, RNA polymerase.

In optical tweezers, DNA and RNAP are tethered to a polystyrene bead and trapped at the center of a focused laser beam, where a strong electric field pulls the bead toward the center. The trap system can be described as a simple linear spring that follows the linear Hooke’s law within a short distance to the center of the beam (55). According to the Hooke’s law, *F = kx*, where *F* is optical force (piconewton level) and *x* is the displacement from center of the beam (nanometer level); optical tweezers enable controlling the force to measure the stiffness of the system (Δ*x*). The force generated from the system can be measured by the displacement. Therefore, optical tweezers can measure kinetic steps of the RNAP. In the single-trap system, one end of the DNA fragment is immobilized on the surface, whereas the rest is held by the RNAP complex attached to a trapped polystyrene bead (Fig. 7*A*). The dual-trap tweezers tether both DNA and RNAP on two beads trapped by two separate laser beams. This arrangement significantly reduces the fluctuation noise and thereby resolves single base pair distance change (Fig. 7*B*). The bead-to-bead distance can be used as a reference scaler to calibrate the displacement of the sample (135). The distance between the beads changes as the RNAP moves on the DNA template through the elongation cycle. With proper calibration between the bead-to-bead distance and the corresponding length of DNA in base pairs, the translocation rate of the EC was calculated from the real-time single-molecule traces (27, 28).

Since optical tweezers can generate the real-time extension traces, the active translocation (on-path) and RNAP pausing (off-path) can be distinguished (Fig. 7*C*) (29, 77, 136, 137). As expected, the transcription through nucleosomes reflected a barrier effect evidenced by pauses in RNAP translocation under varying ionic strength (138). Furthermore, the formation of terminator hairpin (95) and riboswitch structure (96) causes stalling of RNAP or bypass synthesis, which can be distinguished from the real-time trace. In addition, another type of optical trap, angular optical trap, which tethers the DNA molecule by a nanofabricated quartz cylinder instead of bead, can measure the rotation, torque, displacement and force simultaneous. Angular optical trap extends the application of optical tweezers in studying RNAP pausing under torsion (139). Later, the binding and translocation of the Rho factor during termination which induced contour length change of mRNA by a looping conformation was successfully detected (97).

#### Magnetic tweezers

**Figure 8:**
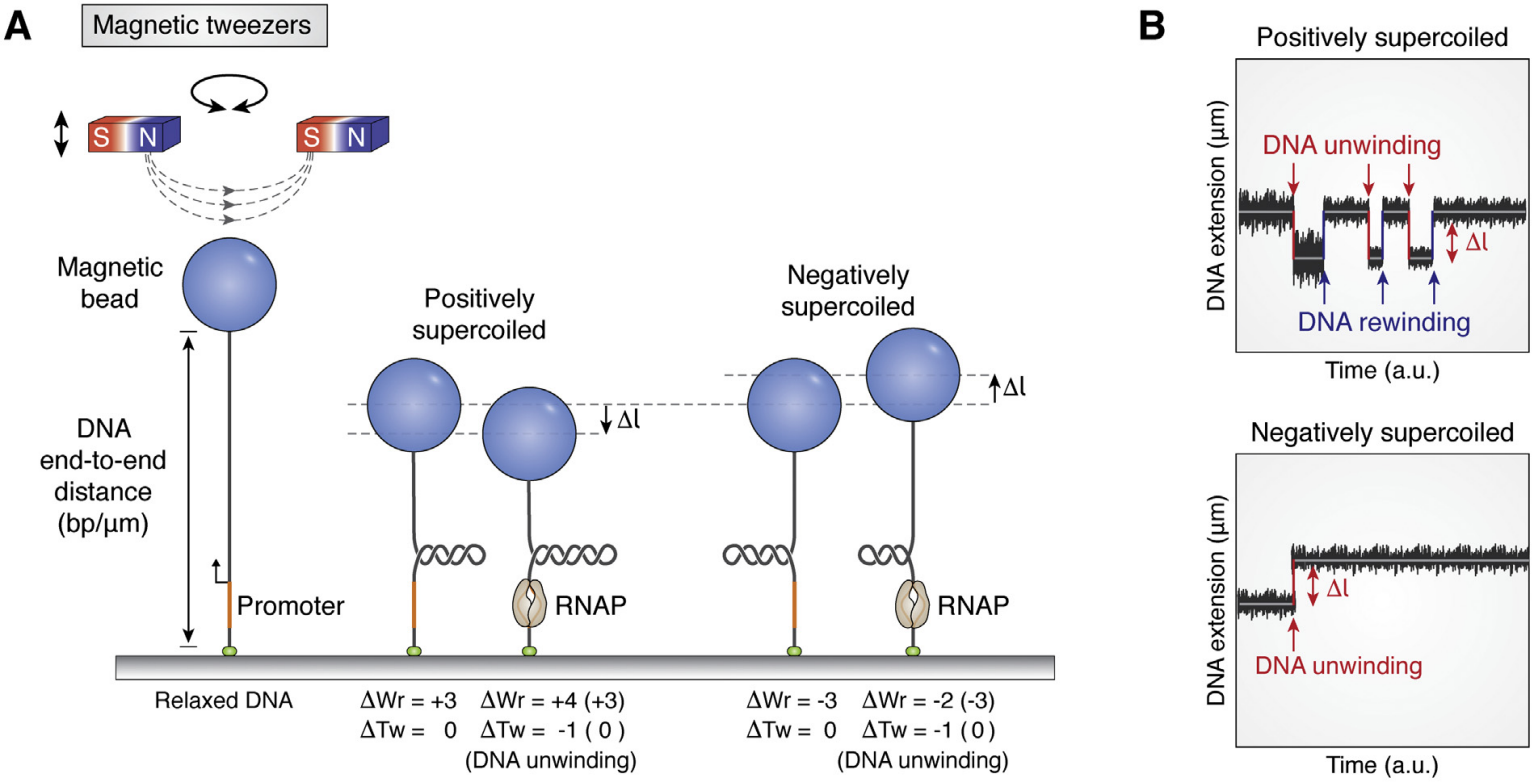
**Transcription initiation measured by magnetic tweezers.***A*, scheme of a magnetic tweezer and DNA topologies. Unwinding DNA in the RP_o_ complex causes the loss of twist, which is later compensated by the writhe. Magnetic tweezers detect the topological change whenever the end-to-end distance of the tethered DNA is shortened or lengthened (Δ*l*) during DNA unwinding. *B*, the examples of real-time traces with positively and negatively supercoiled DNA substrates. Opening the positively supercoiled DNA is an unstable and reversible transition, which can be observed in repeated unwinding (*red arrow*) and rewinding (*blue arrow*) signals. In contrast, opening the negatively supercoiled DNA is a stable and irreversible transition, resulting in one unwinding (*red arrow*) event during the initiation. The schematics are inspired by Revyakin’s work (30). RPo, RNAP–promoter open complex.

It is well-known that RNAP causes the change in DNA supercoiling during transcription. Therefore, DNA supercoiling is also a useful proxy correlated with the transcription process. The suitable tool to measure DNA supercoiling is the magnetic tweezers, a magnetic trapping microscopy that controls the strength and the orientation of the sample by using a magnetic field (19). In this method, one end of the DNA is tethered on the surface, whereas the other end is tagged to a superparamagnetic bead and trapped by a magnet. Therefore, the trapped DNA is unable to drift or rotate, except by the Brownian motion of the bead, which is used to calibrate the force and end-to-end distance of the DNA (Fig. 8*A*). Magnetic tweezers apply force to move the bead vertically away from the surface to stretch the DNA and reduce the noise from thermal fluctuations. In addition, the DNA supercoiling is constrained by restricting the rotation of the bead to fix the DNA topology, specifically the linking number (Lk). This number describes the correlation of the twist (Tw) and the writhe (Wr) of the DNA topology, that is, ΔLk = ΔTw + ΔWr (140). Twist is the number of turns within the dsDNA, and writhe is the number of intramolecular dsDNA crossings. The constrained DNA fragment with a positive or negative ΔLk is referred to as positive or negative supercoiling of DNA. Importantly, the Lk of a torsionally constrained DNA is unable to change (ΔLk = 0), which means that the gain of twist must be compensated by the loss of writhe and vice versa (ΔTw = −ΔWr). Because the writhe indicates the structural crossover within a single DNA, the change of writhe drastically alters the DNA end-to-end distance (141).

In the context of DNA unwinding during initiation, DNA can be induced to a positive or negative writhe (ΔWr > 0 or ΔWr < 0) by rotating the bead of the magnetic tweezers set up. Upon RNAP binding, the closed form complex (RPc) transits to the open form complex (RPo) *via* unwinding the dsDNA about one turn (ΔTw = −1). For example, if ΔWr = +3, loss of ΔTw increases ΔWr to +4, leading to DNA compaction; in contrast, if ΔWr = −3, loss of ΔTw increases ΔWr to −2, resulting in the DNA relaxation by one writhe and the corresponding extension (Fig. 8*A*). Therefore, the unwinding can be detected by measuring the DNA extension (Fig. 8*B*). Furthermore, the kinetics of unwinding and the size of transcription bubble can be observed by the real-time measurement of the change of DNA extension (30, 31, 53). Although magnetic tweezers cannot depict the relative distance change, it can study the kinetics of each transcription stage if supercoiling changes. For example, the comparison of the transcription bubble size between the initiation complex and EC (53) and the unwinding by helicase that facilitated termination was demonstrated (98).

### AFM

**Figure 9:**
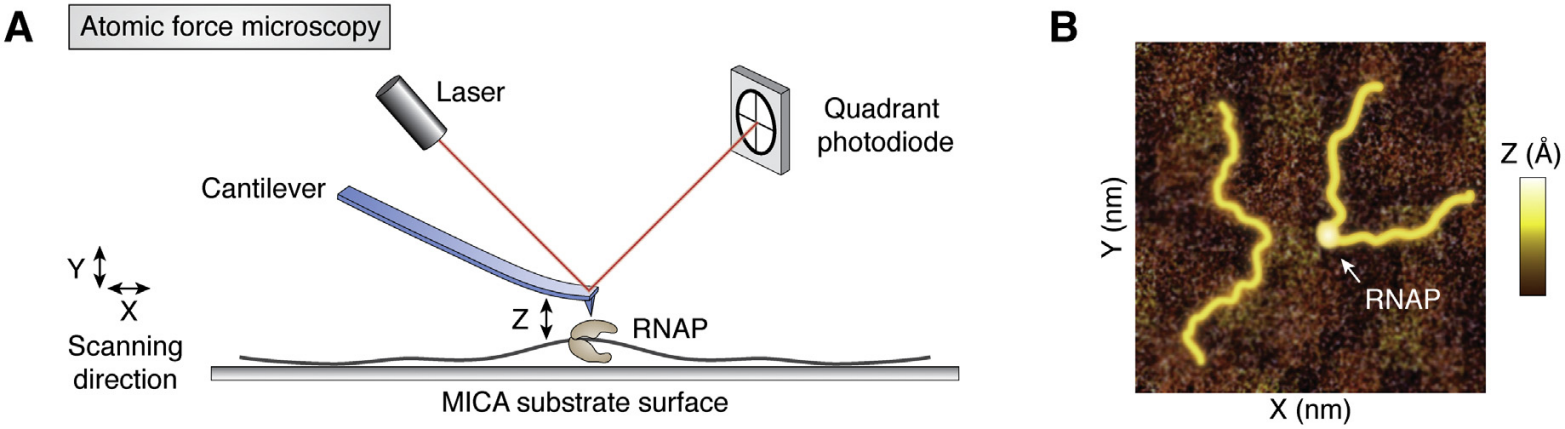
**Atomic force microscopy.***A*, scheme of the AFM. AFM scans the height (Z) of 2D surface (XY) to create a molecular altitude map and visualize the shape of molecule. *B*, an example of the AFM image. The *white arrow* indicates a higher Z-axis value, which is due to RNAP binding. RNAP, RNA polymerase.

AFM, a scanning probe microscopy, allows for a direct visualization of molecules with a high spatial resolution. AFM reveals the three-dimensional shape (topography) of a molecule by scanning the surface and recording the height of a probe tip that depends on the protrusions of the surface-bound molecules (18, 142) (Fig. 9*A*). Samples for AFM do not require a harsh chemical treatment, making it less disruptive to the biological properties. Conventional AFM provides single-digit nanometer resolution but is limited by the low scanning speeds. However, recent advancements in AFM allow for a drastically improved scanning rate of subsecond (2 frame/s) while maintaining the same degree of spatial resolution (35). This high-speed AFM was applied to visualize the RNAP–DNA interaction. Briefly, DNA was mixed with RNAP and deposited on a mica disc in an imaging buffer. The scanned image displayed the RNAP binding as a bright dot, commensurate with the three-dimensional volume of RNAP that changes the vertical height of the probing tip from the surface (Fig. 9*B*). The trajectory of RNAP movement was reconstructed by combining series frames of scanning images as a time-lapse video.

AFM is well suited for measuring DNA structural changes, for example, bending and wrapping of DNA by RNAP. Bending and wrapping during initiation was proposed based on footprinting data (61, 143, 144). AFM images directly confirmed the wrapped state of DNA with a bending angle and a shorter contour length of DNA template upon RNAP binding to the promoter sequence. These images also revealed the transition states, RPc and RPo as well as a looped structure in a complex with a TF (34). Furthermore, a recent study used fast-scan AFM to capture RNAP sliding, hopping, and dissociation and re-association on DNA (through random walk on the mica surface) (35). Interestingly, AFM enables observation of intersegmental transfer of RNAP, which cannot be reliably deduced by other single-molecule methods. One drawback of the AFM measurement is that the molecules tethered to the mica surface may impact the protein conformation or activity.

## Conclusion

Investigating transcription regulation is essential to understand how gene expression is controlled. Transcription of housekeeping genes *versus* regulatory genes and oncogenes *versus* tumor suppressor genes can operate under vastly different regulating conditions exhibiting diverse types of stochastic transcription. One important aspect of these processes is the underlying kinetics and mechanism governed by the RNAP activity. Single-molecule methods provide highly accurate structure–function dynamics in transcription with high temporal and spatial resolution (millisecond, nanometer). Here, we summarize the recent usage of single-molecule techniques by categorizing them into four classes, fluorescence-based microscopy, optical tweezers, magnetic tweezers, and AFM-based detection, and describe the practical application to probe the transcription stages. We note that these methods can also be coupled to other systems, for example, optical tweezers coupled with a fluorescence microscopy can detect the mechanical force and visualize the molecule translocation simultaneously (145, 146, 147). Similarly, the magnetic tweezers coupled with smFRET can measure the RNAP dynamics as well as the topological changes of DNA (148). In addition, other single-molecule sequencing methods have the potential to contribute to studies of transcription. For instance, single-molecule picometer-resolution nanopore tweezers read out the DNA sequence by the electron current change when it passes through a protein-formed nanopore (149). Similarly, a solid-state nanopore was used to detect the RNAP–DNA complex by measuring the change of conductance (150).

To date, these techniques have demonstrated their ability to study *in vitro* transcription in simple systems, such as viral and bacterial cases. However, for a decade, single-molecule transcription studies have reached a bottleneck because of the complexity of the eukaryotic system. Eukaryotic transcription, which is regulated by lots of TFs and undergoes a slow, yet dynamic assembly process, has challenged scientists in investigating the complex transcription components and mechanisms. We look forward to seeing adaptation of single-molecule approaches by researchers outside of the biophysics community to deepen our knowledge about the transcriptional regulation.

## Conflict of interest

The authors declare that they have no conflicts of interest with the contents of this article.
